# Supplementary material for: The RND efflux system ParXY affects siderophore secretion in Pseudomonas putida KT2440
Source: Microbiol Spectr. 2023 Oct 6;11(6):e02300-23. doi: 10.1128/spectrum.02300-23 (PMC10715066; doi:10.1128/spectrum.02300-23)
Supplement: Supplemental material — Tables S1 to S3 and Fig. S1 to S6. [file spectrum.02300-23-s0001.pdf]

Supplementary Information

The RND efflux system ParXY affects pyoverdine secretion in *Pseudomonas putida* KT2440.

Nicola Victoria Stein[1], Michelle Eder[1], Fabienne Burr[1], Sarah Stoss[1*], Lorenz Holzner[2], Hans-Henning Kunz[2] and Heinrich Jung[1#]

[1]Microbiology, Faculty of Biology, Ludwig Maximilians University Munich, Großhaderner Straße 2-4, D-82152 Martinsried, Germany

[2]Plant Biochemistry, Faculty of Biology, Ludwig Maximilian University Munich, Großhaderner Straße 2-4, D-82152 Martinsried, Germany

[#]Address correspondence to H. Jung, hjung@lmu.de, Phone: +49-89-2180-74630

*Present address: Technical University Munich, Arcisstraße 21, D-80333 Munich, Germany

Materials included:

Table S1. Strains used in this investigation.

Table S2. Plasmids used in this investigation.

Table S3. Oligonucleotides used in this investigation.

Figure S1: Alignment of ParX of *P. putida* KT2440 and selected homologs.

Figure S2: Impact of the deletion of *parX* on growth of *P. putida* KT2440 in the presence of chloramphenicol.

Figure S3: Impact of the deletion of *parX* on colony morphology.

Figure S4. Growth rate for complementation of Δ*pm*Δ*parX* phenotype in CAA medium.

Figure S5: Secreted pyoverdine in the supernatant after 24 h in CAA medium.

Figure S6: Effect of $FeCl_3$ addition on the growth of different mutants in KB medium supplemented with 2'2-bipyridyl.

Supplementary References

Table S1. Strains used in this investigation.

Strain	Description	Source/Reference
Escherichia coli DH5α	F^- endA1 glnV44 *thi*-1 *recA*1 *relA*1 *gyrA96 deoR nupG* Φ80d*lacZ*ΔM15 Δ(*lacZYA-argF*)U169, *hsdR17*(r_K^- m_K^+), λ–	[1, 2]
Escherichia coli WM3064	thrB1004 *pro thi rpsL hsdS lacZ*ΔM15 RP4-1360 Δ(*araBAD*) 567 Δ*dapA*1341::[erm pir]	W. Metcalf, Univ. of Illinois, Urbana
Pseudomonas putida KT2440	wild- type strain	[3, 4]
Pseudomonas putida KT2440R ΔT6SS	Markerless KT2440R null mutant in the *tssA1/tssM2/tssM3*	[2]
Pseudomonas putida KT2440 Δ*pvd*	Δp*vdRT-opmQ* (PP_4209-PP_4211)	[5]
Pseudomonas putida KT2440 *Δmdt*	*ΔmdtA* (PP_3585)	This work
Pseudomonas putida KT2440 Δ*parX*	Δ*parX* (PP_3455)	This work
Pseudomonas putida KT2440 Δ*pm*	Δp*vdRT-opmQ* (PP_4209-PP_4211) Δ*mdtA* (PP_3585)	[6]
Pseudomonas putida KT2440 Δ*pvd*Δ*parX*	Δp*vdRT-opmQ* (PP_4209-PP_4211) Δ*parX* (PP_3455)	This work
Pseudomonas putida KT2440 Δ*mdt*Δ*parX*	Δ*mdtA* (PP_3585) Δ*parX* (PP_3455)	This work
Pseudomonas putida KT2440 Δ*pm*Δ*parX*	Δp*vdRT-opmQ* (PP_4209-PP_4211) Δ*mdtA* (PP_3585) Δ*parX* (PP_3455)	This work

Table S1

Pseudomonas putida KT2440 Δ*pm*Δ*pp0166*	Δ*pvdRT-opmQ* (PP_4209-PP_4211) Δ*mdtA* (PP_3585) Δ*paxA* (PP_0166)	This work
Pseudomonas putida KT2440 Δ*pm*Δ*pp0804*	Δ*pvdRT-opmQ* (PP_4209-PP_4211) Δ*mdtA* (PP_3585) Δ*pp0804* (PP_0804)	This work
Pseudomonas putida KT2440 Δ*pm*Δ*pp0906*	Δ*pvdRT-opmQ* (PP_4209-PP_4211) Δ*mdtA* (PP_3585) Δ*mexV* (PP_0906)	This work
Pseudomonas putida KT2440 Δ*pm*Δ*pp1264*	Δ*pvdRT-opmQ* (PP_4209-PP_4211) Δ*mdtA* (PP_3585) Δ*fusBCD* (PP_1264)	This work
Pseudomonas putida KT2440 Δ*pm*Δ*pp1516*	Δ*pvdRT-opmQ* (PP_4209-PP_4211) Δ*mdtA* (PP_3585) Δ*mexJ* (PP_1516)	This work
Pseudomonas putida KT2440 Δ*pm*Δ*pp2064*	Δ*pvdRT-opmQ* (PP_4209-PP_4211) Δ*mdtA* (PP_3585) Δ*mexH* (PP_2064)	This work
Pseudomonas putida KT2440 Δ*pm*Δ*pp2818*	Δ*pvdRT-opmQ* (PP_4209-PP_4211) Δ*mdtA* (PP_3585) Δ*mexD* (PP_2818)	This work
Pseudomonas putida KT2440 Δ*pm*Δ*pp3426*	Δ*pvdRT-opmQ* (PP_4209-PP_4211) Δ*mdtA* (PP_3585) Δ*mexF* (PP_3426)	This work
Pseudomonas putida KT2440 Δ*pm*Δ*pp3549*	Δ*pvdRT-opmQ* (PP_4209-PP_4211) Δ*mdtA* (PP_3585) Δ*emrA* (PP_3549)	This work
Pseudomonas putida KT2440 Δ*pm*Δ*pp5173*	Δ*pvdRT-opmQ* (PP_4209-PP_4211) Δ*mdtA* (PP_3585) Δ*triC* (PP_5173)	This work
Pseudomonas putida KT2440 Δ*pm*Δ*pp1019*	Δ*pvdRT-opmQ* (PP_4209-PP_4211) Δ*mdtA* (PP_3585) Δ*oprB-I* (PP_1019)	This work

Table S1

Pseudomonas putida KT2440 Δ*pm*Δ*pp1273*	Δ*pvdRT-opmQ* (PP_4209-PP_4211) Δ*mdtA* (PP_3585) Δ*pp1273* (PP_1273)	This work
Pseudomonas putida KT2440 Δ*pm*Δ*pp1798*	Δ*pvdRT-opmQ* (PP_4209-PP_4211) Δ*mdtA* (PP_3585) Δ*pp1798* (PP_1798)	This work
Pseudomonas putida KT2440 Δ*pm*Δ*pp2069*	Δ*pvdRT-opmQ* (PP_4209-PP_4211) Δ*mdtA* (PP_3585) Δ*pp2069* (PP_2069)	This work
Pseudomonas putida KT2440 Δ*pm*Δ*pp2558*	Δ*pvdRT-opmQ* (PP_4209-PP_4211) Δ*mdtA* Δ*pp2558* (PP_2558)	This work
Pseudomonas putida KT2440 Δ*pm*Δ*pp3427*	Δ*pvdRT-opmQ* (PP_4209-PP_4211) Δ*mdtA* (PP_3585) Δ*oprN* (PP_3427)	This work
Pseudomonas putida KT2440 Δ*pm*Δ*pp4519*	Δ*pvdRT-opmQ* (PP_4209-PP_4211) Δ*mdtA* (PP_3585) Δ*pp4519* (PP_4519)	This work
Pseudomonas putida KT2440 Δ*pm*Δ*pp4923*	Δ*pvdRT-opmQ* (PP_4209-PP_4211) Δ*mdtA* (PP_3585) Δ*pp4923* (PP_4923)	This work
Pseudomonas putida KT2440R Δ*pm*ΔT6SS	Δ*pvdRT-opmQ* (PP_4209-PP_4211) Δ*mdtA* (PP_3585) Δ*tssA1* Δ*tssM2* Δ*tssM3*	This work
Pseudomonas putida KT2440 3E2	PP_4220*::*Tn5, pyoverdine negative Tn5 mutant of *P. putida*, TcR	[7]

Table S2. Plasmids used in this investigation.

Plasmid	Description	Source/Reference
pNPTS-138-R6KT	Suicide plasmid for in-frame deletions; *mobRP4+, oriR6K sacB KmR*	[8]
pSEVA224	KmR; pSEVA221-derivative with *lacIq/Ptrc* expression system	[9]
pSEVA224-*parX*	pSEVA224 derivative with insertion of PP_3455 in the multiple cloning site	This work
pBBR1-MCS5-*lux*	*luxCDABE*, empty plasmid, with *aacC1* gene (GenR)	[10]
pBBR1-Ppp4243-lux	pBBR1-MCS5-lux derivative with insertion of promoter region upstream of pp_4243	[11]
pBBR1-Ppp4209-*lux*	pBBR1-MCS5-lux derivative with insertion of promoter region upstream of pp_4209	[5]
pBBR1-Ppp3585-*lux*	pBBR1-MCS5-lux derivative with insertion of promoter region upstream of pp_3585	[5]
pBBR1-Ppp3455-*lux*	pBBR1-MCS5-lux derivative with insertion of promoter region upstream of pp_3455	This work

Table S3. Oligonucleotides used in this investigation. Restriction sites are marked in red.

Oligonucleotides	Sequence (5' ->3')
Ppp_4243_300bp_fwd	CTGCAGGAATTCTCACCTTGTCACAGAGGGGA
Ppp_4243_300bp_rev	ATCGATGGGCCCTGGGTGTTCCTGCCAGTGTC
Ppp_3585_300bp_fwd	CTGCAGGAATTCATTGACCCCGCGCGCCTGTT
Ppp_3585_300bp_rev	ATCGATAAGCTTGGGTTGAACGAACCTTGTTC
lux_4209_s	GGACCCGAATTCAAGCATCGTGAGCGACGTCC
lux_4209_as	TGAGCGAAGCTTGCGGACTTCCAGCCAGGGAT
Ppp3455short_BamHI-fw	TAGAACGGATCCACTCGACA
Ppp3455_XhoI_rv	GCCTCGAGATCTTCCGAT
PP_3455-fw	ATCTCTAGATCCATATGCCTACT
PP_3455-rv	ATGAAGCTTGCCCTCACTG
**Oligos for sequencing**	
pBBR sense2	GTAGGACAAATCCGCCGCTAGG
pUC19s	AAGTTGGGTAACGCCAGGGT
M13 Fw(-41)	GGTTTTCCCAGTCACGAC
Seq pp3455 end	TGCTGGTGGTAGAC
Seq pp3455 start	TCACGGTGGCATAG
seq_pSEVA224_as2	TCATTACTGGATCTATCAACAGGAG
Seq_pp3455_bp1120_rev	TTGTCGACCTGCACCT
pUCP-NdeI-MCS-R	GAGCGGATAACAATTTCACA
pUCP-NdeI-MCS-RII	TCATTAATGCAGCTGGCAC

Figure S1

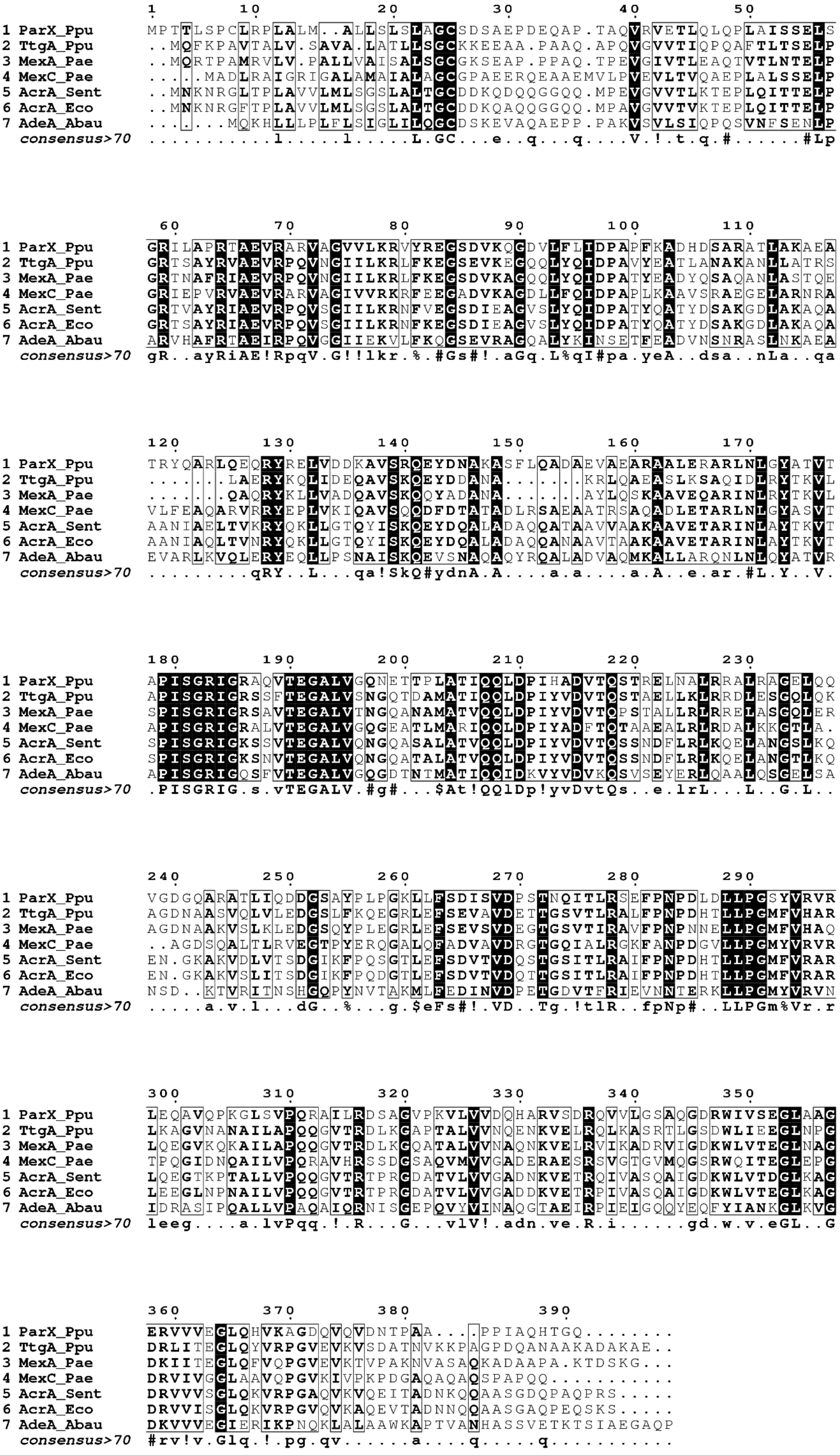
1 10 20 30 40 50
1 ParX_Ppu MPTTLSPCLRPLALM..ALLSLSLAGCSDSAEPDEQAP.TAQVRVETLQLQPLAISSELS
2 TtgA_Ppu ..MQFKPAVTALV.SAVA.LATLLSGCKKEEAA.PAAQ.APQVGVVTIQPQAFTLTSELP
3 MexA_Pae ..MQRTPAMRVLV.PALLVAISALSGCGKSEAP.PPAQ.TPEVGIVTLEAQTVTLNTELP
4 MexC_Pae .....MADLRAIGRIGALAMAIALAGCGPAEERQEAAEMVLPVEVLTVQAEPLALSSELP
5 AcrA_Sent ..MNKNRGLTPLAVVLMLSGSLALTGCDDKQDQQGGQQ.MPEVGVVTLKTEPLQITTELP
6 AcrA_Eco ..MNKNRGFTPLAVVLMLSGSLALTGCDDKQAQQGGQQ.MPAVGVVTVKTEPLQITTELP
7 AdeA_Abau .......MQKHLLLPLFLSIGLILQGCDSKEVAQAEPP.PAKVSVLSIQPQSVNFSENLP
consensus>70 ....l.....l.....L.GC...e..q...q....V.!.t.q.#......#Lp
60 70 80 90 100 110
1 ParX_Ppu GRILAPRTAEVRARVAGVVLKRVYREGSDVKQGDVLFLIDPAPFKADHDSARATLAKAEA
2 TtgA_Ppu GRTSAYRVAEVRPQVNGIILKRLFKEGSEVKEGQQLYQIDPAVYEATLANAKANLLATRS
3 MexA_Pae GRTNAFRIAEVRPQVNGIILKRLFKEGSDVKAGQQLYQIDPATYEADYQSAQANLASTQE
4 MexC_Pae GRIEPVRVAEVRARVAGIVVRKRFEEGADVKAGDLLFQIDPAPLKAAVSRAEGELARNRA
5 AcrA_Sent GRTVAYRIAEVRPQVSGIILKRNFVEGSDIEAGVSLYQIDPATYQATYDSAKGDLAKAQA
6 AcrA_Eco GRTSAYRIAEVRPQVSGIILKRNFKEGSDIEAGVSLYQIDPATYQATYDSAKGDLAKAQA
7 AdeA_Abau ARVHAFRTAEIRPQVGGIIEKVLFKQGSEVRAGQALYKINSETFEADVNSNRASLNKAEA
consensus>70 gR..ayRiAE!RpqV.G!!lkr.%.#Gs#!.aGq.L%qI#pa.yeA..dsa..nLa..qa
120 130 140 150 160 170
1 ParX_Ppu TRYQARLQEQRYRELVDDKAVSRQEYDNAKASFLQADAEVAEARAALERARLNLGYATVT
2 TtgA_Ppu .......LAERYKQLIDEQAVSKQEYDDANA.......KRLQAEASLKSAQIDLRYTKVL
3 MexA_Pae .......QAQRYKLLVADQAVSKQQYADANA.......AYLQSKAAVEQARINLRYTKVL
4 MexC_Pae VLFEAQARVRRYEPLVKIQAVSQQDFDTATADLRSAEAATRSAQADLETARLNLGYASVT
5 AcrA_Sent AANIAELTVKRYQKLLGTQYISKQEYDQALADAQQATAAVVAAKAAVETARINLAYTKVT
6 AcrA_Eco AANIAQLTVNRYQKLLGTQYISKQEYDQALADAQQANAAVTAAKAAVETARINLAYTKVT
7 AdeA_Abau EVARLKVQLERYEQLLPSNAISKQEVSNAQAQYRQALADVAQMKALLARQNLNLQYATVR
consensus>70 ....qRY..L...qa!SkQ#ydnA.A....a.a....a.A..e.ar.#L.Y..V.
180 190 200 210 220 230
1 ParX_Ppu APISGRIGRAQVTEGALVGQNETTPLATIQQLDPIHADVTQSTRELNALRRALRAGELQQ
2 TtgA_Ppu APISGRIGRSSFTEGALVSNGQTDAMATIQQLDPIYVDVTQSTAELLKLRRDLESGQLQK
3 MexA_Pae SPISGRIGRSAVTEGALVTNGQANAMATVQQLDPIYVDVTQPSTALLRLRRELASGQLER
4 MexC_Pae APISGRIGRALVTEGALVGQGEATLMARIQQLDPIYADFTQTAAEALRLRDALKKGTLA.
5 AcrA_Sent SPISGRIGKSSVTEGALVQNGQASALATVQQLDPIYVDVTQSSNDFLRLKQELANGSLKQ
6 AcrA_Eco SPISGRIGKSNVTEGALVQNGQATALATVQQLDPIYVDVTQSSNDFLRLKQELANGTLKQ
7 AdeA_Abau APISGRIGQSFVTEGALVGQGDTNTMATIQQIDKVYVDVKQSVSEYERLQAALQSGELSA
consensus>70 .PISGRIG.s.vTEGALV.#g#...$At!QQlDp!yvDvtQs..e.lrL...L..G.L..
240 250 260 270 280 290
1 ParX_Ppu VGDGQARATLIQDDGSAYPLPGKLLFSDISVDPSTNQITLRSEFPNPDLDLLPGSYVRVR
2 TtgA_Ppu AGDNAASVQLVLEDGSLFKQEGRLEFSEVAVDETTGSVTLRALFPNPDHTLLPGMFVHAR
3 MexA_Pae AGDNAAKVSLKLEDGSQYPLEGRLEFSEVSVDEGTGSVTIRAVFPNPNNELLPGMFVHAQ
4 MexC_Pae ..AGDSQALTLRVEGTPYERQGALQFADVAVDRGTGQIALRGKFANPDGVLLPGMYVRVR
5 AcrA_Sent EN.GKAKVDLVTSDGIKFPQSGTLEFSDVTVDQSTGSITLRAIFPNPDHTLLPGMFVRAR
6 AcrA_Eco EN.GKAKVSLITSDGIKFPQDGTLEFSDVTVDQTTGSITLRAIFPNPDHTLLPGMFVRAR
7 AdeA_Abau NSD..KTVRITNSHGQPYNVTAKMLFEDINVDPETGDVTFRIEVNNTERKLLPGMYVRVN
consensus>70 .....a.v.l...dG..%...g.$eFs#!.VD..Tg.!tlR..fpNp#..LLPGm%Vr.r
300 310 320 330 340 350
1 ParX_Ppu LEQAVQPKGLSVPQRAILRDSAGVPKVLVVDQHARVSDRQVVLGSAQGDRWIVSEGLAAG
2 TtgA_Ppu LKAGVNANAILAPQQGVTRDLKGAPTALVVNQENKVELRQLKASRTLGSDWLIEEGLNPG
3 MexA_Pae LQEGVKQKAILAPQQGVTRDLKGQATALVVNAQNKVELRVIKADRVIGDKWLVTEGLNAG
4 MexC_Pae TPQGIDNQAILVPQRAVHRSSDGSAQVMVVGADERAESRSVGTGVMQGSRWQITEGLEPG
5 AcrA_Sent LQEGTKPTALLVPQQGVTRTPRGDATVLVVGADNKVETRQIVASQAIGDKWLVTDGLKAG
6 AcrA_Eco LEEGLNPNAILVPQQGVTRTPRGDATVLVVGADDKVETRPIVASQAIGDKWLVTEGLKAG
7 AdeA_Abau IDRASIPQALLVPAQAIQRNISGEPQVYVINAQGTAEIRPIEIGQQYEQFYIANKGLKVG
consensus>70 leeg....a.lvPqq.!.R...G...vlV!.adn.ve.R.i......gd.w.v.eGL..G
360 370 380 390
1 ParX_Ppu ERVVVEGLQHVKAGDQVQVDNTPAA....PPIAQHTGQ........
2 TtgA_Ppu DRLITEGLQYVRPGVEVKVSDATNVKKPAGPDQANAAKADAKAE..
3 MexA_Pae DKIITEGLQFVQPGVEVKTVPAKNVASAQKADAAPA.KTDSKG...
4 MexC_Pae DRVIVGGLAAVQPGVKIVPKPDGAQAQAQSPAPQQ...........
5 AcrA_Sent DRVVVSGLQKVRPGAQVKVQEITADNKQQAASGDQPAQPRS.....
6 AcrA_Eco DRVVISGLQKVRPGVQVKAQEVTADNNQQAASGAQPEQSKS.....
7 AdeA_Abau DKVVVEGIERIKPNQKLALAAWKAPTVANHASSVETKTSIAEGAQP
consensus>70 #rv!v.Glq.!.pg.qv......a....q.....q..........

Figure S1

Figure S1: Alignment of ParX of *P. putida* KT2440 and selected homologs. Sequences were taken from the homology search at string-db.org. Overall coverage can be found in Table S5. Respective strain and protein identifiers are (1) *Pseudomonas putida* KT2440 (PP_3455), (2) *Pseudomonas putida* KT2440 (PP_1386), (3) *Pseudomonas aeruginosa* PAO1 (PA0425), (4) *Pseudomonas aeruginosa* PAO1 (PA4599), (5) *Salmonella enterica* CT18 (16501748), (6) *Escherichia coli* K12 MG1655 (b0463), (7) *Acinetobacter baumannii* (IX87_20160). Equivalent amino acids are highlighted based on identity (black background) and similarity according to their physicochemical properties (bold). Alignment was performed using the multiple sequence alignment tool from Clustal Omega (default settings) [12, 13] and the ESPript 3.0 tool (%Equivalent, 0.7 Global scores, 0.5 Diff. score, coloring B&W) [14].

Figure S2

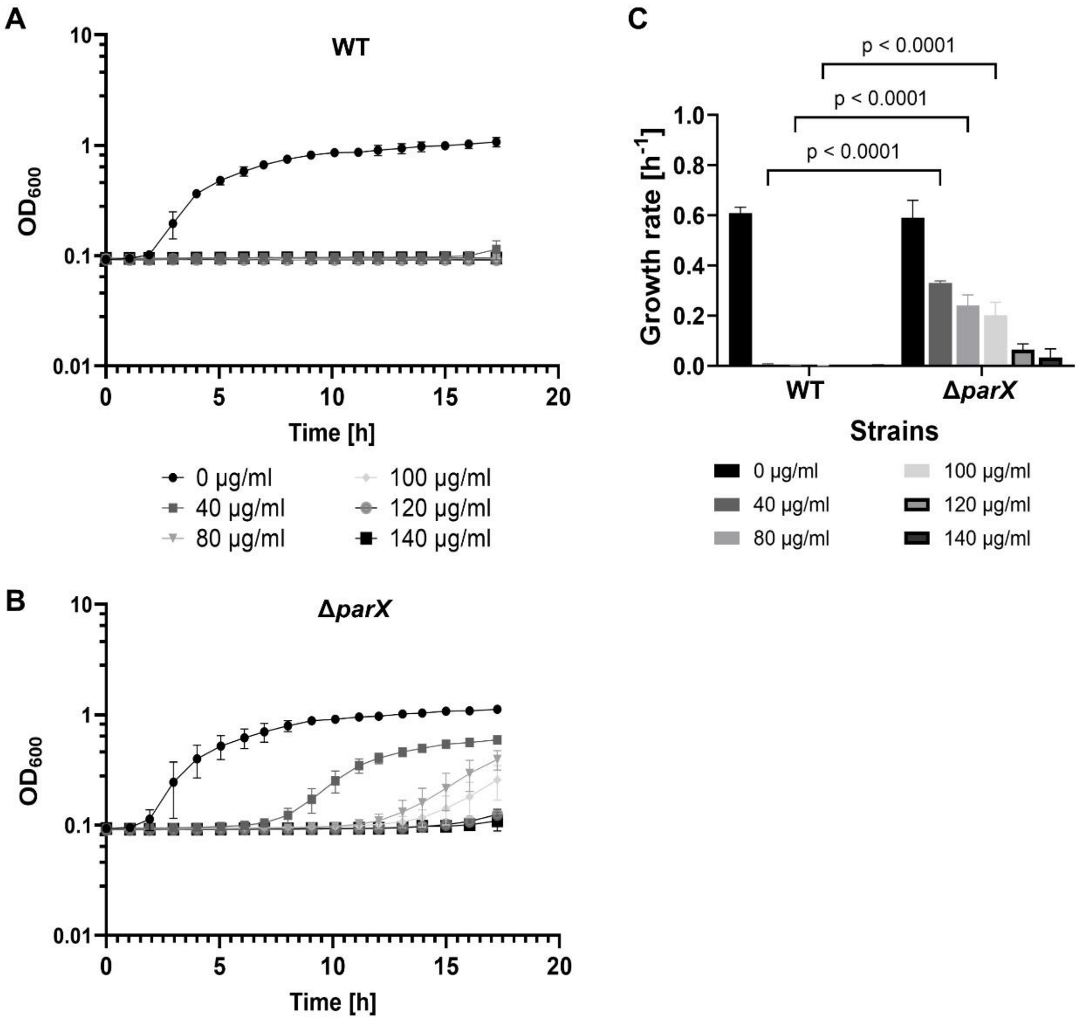

Figure S2. Impact of the deletion of *parX* on growth of *P. putida* KT2440 in the presence of chloramphenicol. (A) *P. putida* KT2440 **(**WT) and (B) *parX* mutant were grown in MH medium supplemented with the antibiotic chloramphenicol at concentrations of 0, 40, 80, 100, 120, to 140 µg per ml for 17 h. Growth was monitored in a Tecan infinite® M200 Pro plate reader. The graph represents the OD_{600} values for each hour of growth at 30°C and continuous shaking with an orbital amplitude of 2. (C) The growth rates were calculated for WT and *parX* mutant, grown in MH medium supplemented with the antibiotic chloramphenicol (as shown in Fig. 6). Data was calculated for strains in the exponential phase. For statistical analysis, three biological replicates were analyzed using a 2-way ANOVA (Šídák's multiple comparisons tests, α 0.05). Only significant *p*-values are shown.

Figure S3

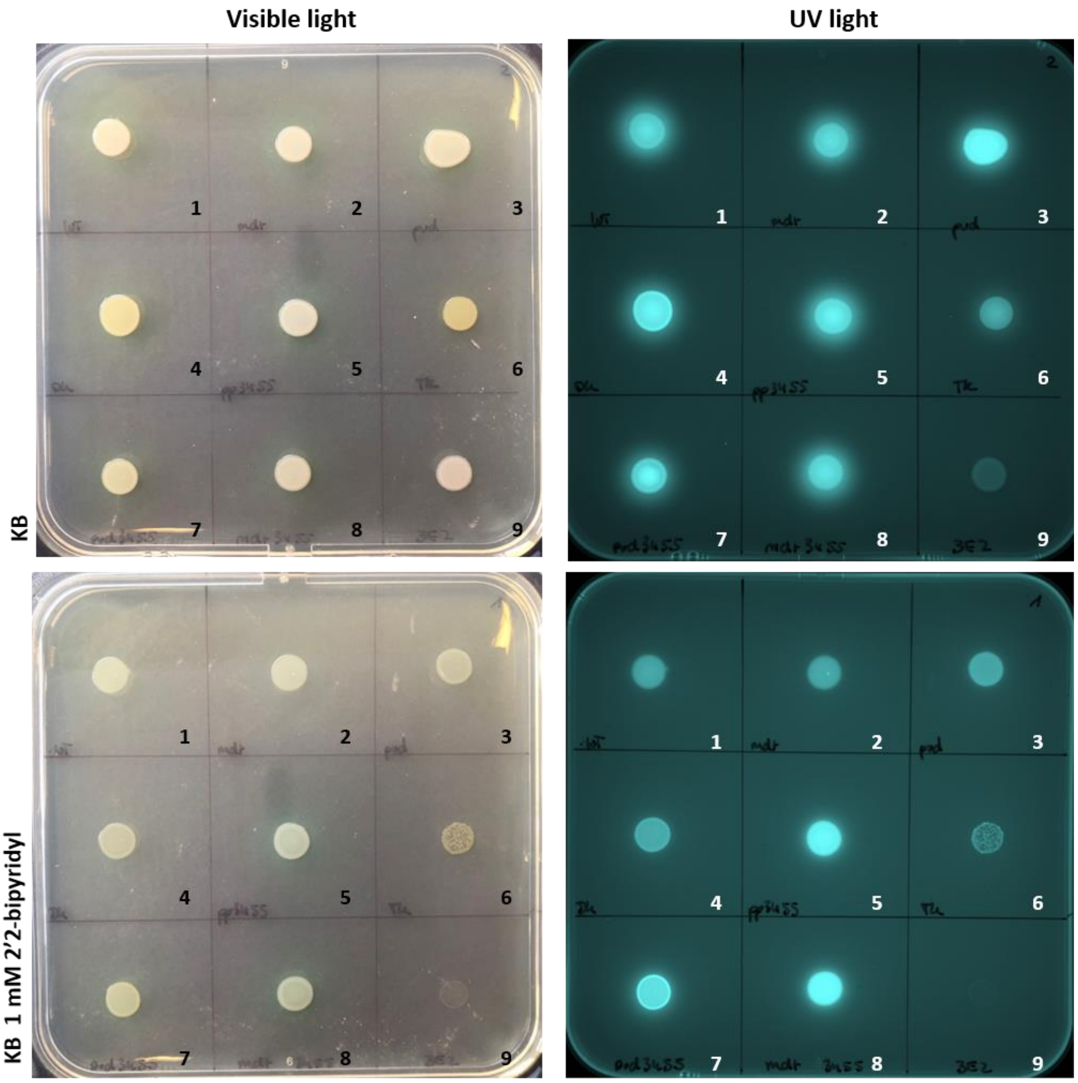

Figure S3: Impact of the deletion of *parX* on colony morphology. Colonies of the wild-type (1), Δ*mdt* (2), Δ*pvd* (3), Δ*pm* (4), Δ*parX* (5), Δ*pm*Δ*parX* (6), Δ*pvd*Δ*parX* (7), Δ*mdt*Δ*parX* (8) and 3E2 (9) on King's B and King's B with 1 mM 2'2 bipyridyl are shown in visible and UV light. Strains are indicated with numbers 1 to 9. Plates were incubated for 18 hours at 30°C and imaged using a BioRad Gel Doc XR+ Gel Documentation System and trans-UV. Figures in the UV channel were stained cyan.

Figure S4

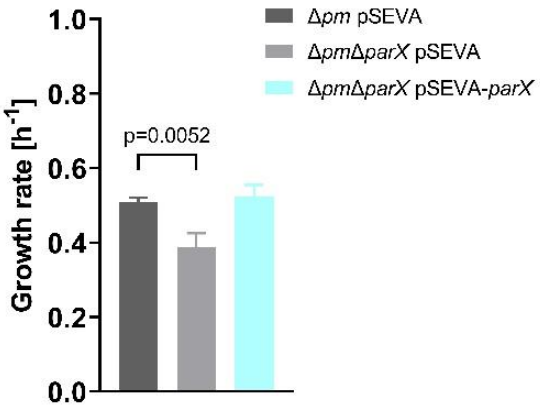

Figure S4: Growth rate for complementation of Δ*pm*Δ*parX* phenotype in CAA medium. Strains containing pSEVA224 plasmids shown in Fig. 4 were grown in a 96-well plate with CAA medium (Fig. 4B) containing 0.5 mM IPTG for gene expression and kanamycin (50 mg per ml) for plasmid maintenance. The growth rate was calculated for strains from Figure 4B in the exponential phase. The triple deletion Δ*pm*Δ*parX* with the complementation plasmid pSEVA-*parX* (cyan) does not differ significantly from the double deletion strain with an empty plasmid (dark grey). In contrast, the triple deletion with the empty plasmid (light grey) differs significantly to both tested strains, with a *p*-value of 0.0052 and 0.0028, respectively. Mean values of a minimum of three biological replicates are shown. For statistical analysis, ANOVA and Tukey's multiple comparisons test (α 0.05) were used.

Figure S5

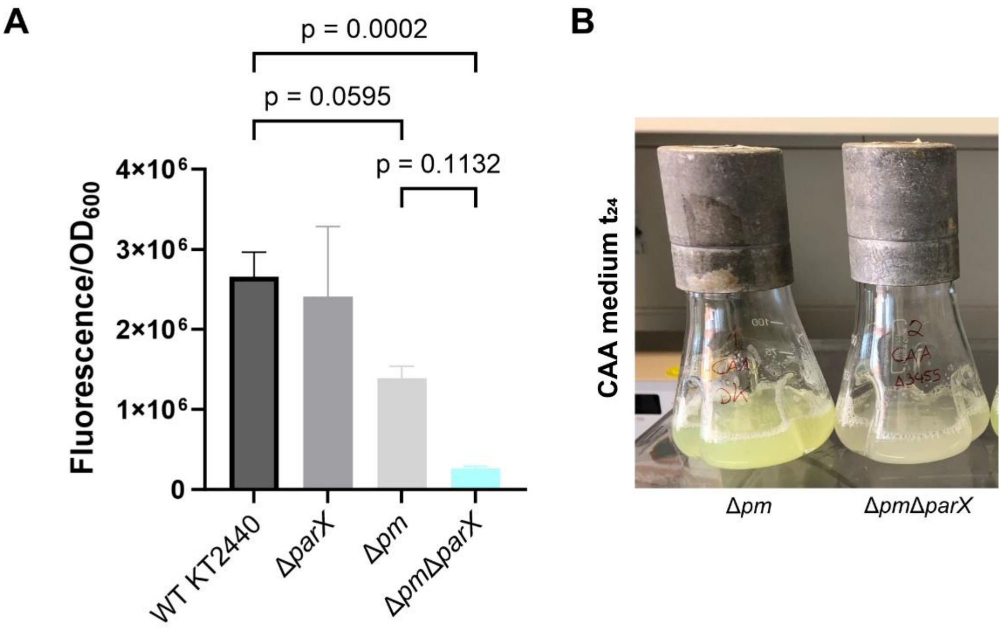

Figure S5: Secreted pyoverdine in the supernatant after 24 h in CAA medium.

(A) Cultures from growth curves in Figure 3C were analyzed for their secreted pyoverdine. Mature pyoverdine in the supernatant was quantified by fluorescence measurements. After 24 hours of growth, 1 ml of culture from each flask was taken and centrifuged (3 min, 15 700 x g). Fluorescence was determined with an excitation wavelength of λ_{ex}: 400 nm and emission wavelength of λ_{em}: 455 nm (Tecan infinite® M200 pro plate reader), respectively. Mean values of a minimum of three biological replicates are shown. ANOVA and Tukey's multiple comparisons test (α 0.05) were used for statistical analysis. (B) Cultures were imaged after 24h of continuous shaking at 30°C. The double deletion Δ*pm* (left) shows more yellow-green pyoverdine than the triple deletion Δ*pm*Δ*parX* (right) by eye.

Figure S6

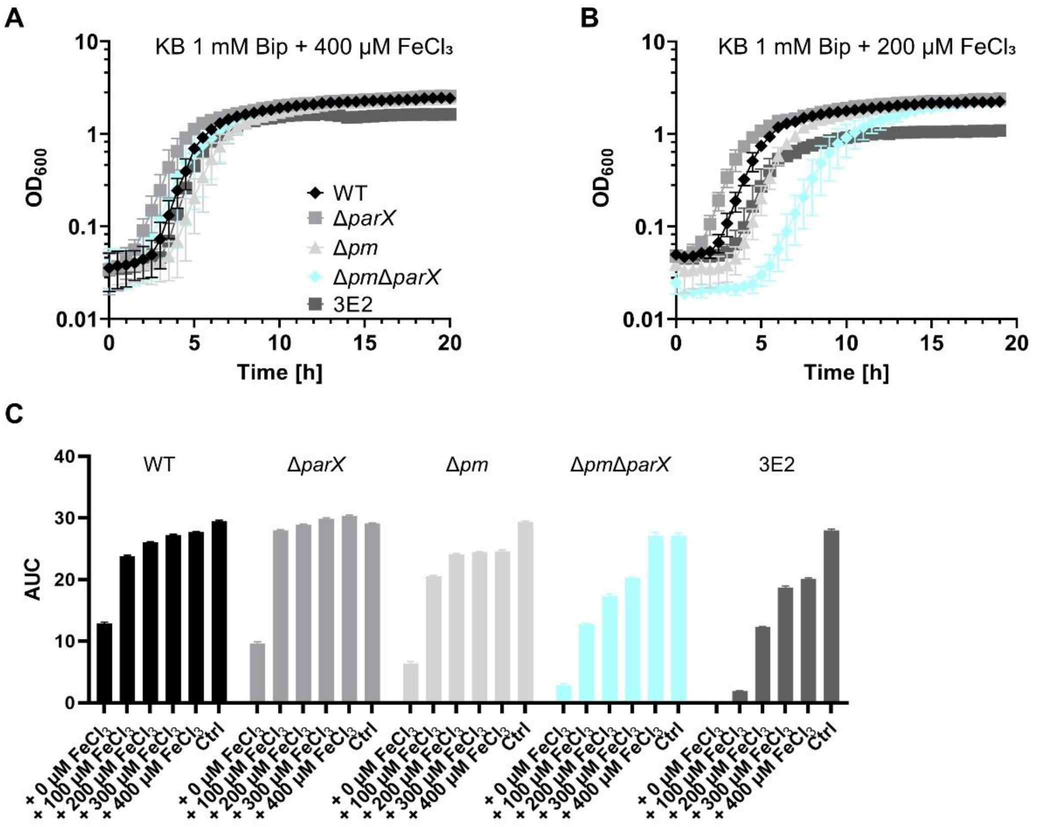

Figure S6: Effect of $FeCl_3$ on the growth of different mutants in KB medium supplemented with 2'2-bipyridyl. KB medium supplemented with 1 mM Bip was inoculated from overnight culture to yield an OD_{600} of 0.05 and incubated as described above. (A) Comparison of growth in KB plus 1 mM Bip supplemented with 400 µM $FeCl_3$ or (B) 200 µM $FeCl_3$. OD_{600} was recorded for 19 h in a CLARIOstar Plus plate reader. (C) The area under the growth curve (AUC) was determined for the conditions from (A) and (B) and various additional conditions (0, 100, and 300 µM $FeCl_3$) and a control medium (ctrl) containing only KB without iron chelator and additional iron. The experiment was performed with at least three biological replicates.

Supplementary References

1. Chen, J., et al., *Whole-genome sequence of phage-resistant strain Escherichia coli DH5alpha.* Genome Announc, 2018. **6**(10).
2. Bernal, P., et al., *The Pseudomonas putida T6SS is a plant warden against phytopathogens.* The ISME Journal, 2017. **11**(4): p. 972-987.
3. Bagdasarian, M., et al., *Specific-purpose plasmid cloning vectors. II. Broad host range, high copy number, RSF1010-derived vectors, and a host-vector system for gene cloning in Pseudomonas.* Gene, 1981. **16**(1-3): p. 237-47.
4. Nelson, K.E., et al., *Complete genome sequence and comparative analysis of the metabolically versatile Pseudomonas putida KT2440.* Environ Microbiol, 2002. **4**(12): p. 799-808.
5. Henriquez, T., N.V. Stein, and H. Jung, *PvdRT-OpmQ and MdtABC-OpmB efflux systems are involved in pyoverdine secretion in Pseudomonas putida KT2440.* Environ Microbiol Rep, 2019. **11**(2): p. 98-106.
6. Stein, N.V., et al., *The ABC transporter family efflux pump PvdRT-OpmQ of Pseudomonas putida KT2440: purification and initial characterization.* FEBS Lett, 2023. **597**(10): p. 1403-1414.
7. Matthijs, S., et al., *Siderophore-mediated iron acquisition in the entomopathogenic bacterium Pseudomonas entomophila L48 and its close relative Pseudomonas putida KT2440.* Biometals, 2009. **22**(6): p. 951-64.
8. Lassak, J., et al., *ArcS, the cognate sensor kinase in an atypical Arc system of Shewanella oneidensis MR-1.* Appl Environ Microbiol, 2010. **76**(10): p. 3263-74.
9. Silva-Rocha, R., et al., *The Standard European Vector Architecture (SEVA): a coherent platform for the analysis and deployment of complex prokaryotic phenotypes.* Nucleic Acids Res, 2013. **41**(Database issue): p. D666-75.
10. Gödeke, J., et al., *Roles of two Shewanella oneidensis MR-1 extracellular endonucleases.* Applied and Environmental Microbiology, 2011. **77**: p. 5342 - 5351.
11. Henriquez, T. and H. Jung, *Involvement of the MxtR/ErdR (CrbS/CrbR) Two-Component System in Acetate Metabolism in Pseudomonas putida KT2440.* Microorganisms, 2021. **9**(8).
12. Sievers, F., et al., *Fast, scalable generation of high-quality protein multiple sequence alignments using Clustal Omega.* Mol Syst Biol, 2011. **7**: p. 539.
13. Goujon, M., et al., *A new bioinformatics analysis tools framework at EMBL–EBI.* Nucleic Acids Research, 2010. **38**(suppl_2): p. W695-W699.
14. Robert, X. and P. Gouet, *Deciphering key features in protein structures with the new ENDscript server.* Nucleic Acids Research, 2014. **42**(W1): p. W320-W324.
